# Indoor Air Pollution and Lung Cancer Risk—A Systematic Review and Meta-Analysis

**DOI:** 10.3390/jcm15051854

**Published:** 2026-02-28

**Authors:** Stefan-Roberto Rusoiu, Norbert Wellmann, Ana Adriana Trusculescu, Andreea Roxana Durdan, Dorotea Carmen Cioanca, Alexandra Bosoanca, Cristian Oancea, Monica Steluta Marc

**Affiliations:** 1 Doctoral School, “Victor Babes” University of Medicine and Pharmacy, 2 Eftimie Murgu Square, RO-300041 Timisoara, Romania; stefan.rusoiu@rezident.umft.ro (S.-R.R.);; 2 Pulmonology University Clinic, Clinical Hospital of Infectious Diseases and Pneumophthisiology “Dr. Victor Babes”, 13 Gheorghe Adam Street, RO-300310 Timisoara, Romania; 3 Center of Research and Innovation in Personalized Medicine of Respiratory Diseases (CRIPMRD), “Victor Babes” University of Medicine and Pharmacy, 2 Eftimie Murgu Square, RO-300041 Timisoara, Romania

**Keywords:** indoor air pollution, lung cancer risk, environmental tobacco smoke, cooking oil fumes, biomass, solid fuel, household exposure

## Abstract

**Background/Objectives**: Indoor air pollution is an increasingly recognized cause of lung cancer, yet evidence remains fragmented across exposure categories. This systematic review aimed to consolidate epidemiological findings on the relationship between household pollutants and lung cancer risk across diverse settings. **Methods**: A systematic search of PubMed, Web of Science, Scopus, and Cochrane was conducted to identify observational studies published between 2015 and 2025. Eligible articles evaluated indoor exposure in relation to primary lung cancer. Maximally adjusted effect estimates were extracted. Random effects models were used to calculate pooled odds ratios (ORs) and hazard ratios (HRs) when appropriate. Study quality was assessed using the Newcastle–Ottawa Scale. **Results**: Thirty-eight studies comprising 475,565 participants were included. Environmental tobacco smoke (ETS) was associated with lung cancer risk (pooled OR 1.97, 95% CI 1.63–2.37; pooled HR 1.44, 95% CI 1.19–1.74). Cooking oil fumes showed a pooled OR of 1.83 (95% CI 1.53–2.21). Solid fuel and biomass combustion were also associated with increased lung cancer risk, with pooled estimates indicating elevated odds and hazard ratios (pooled OR 2.26, 95% CI 1.36–3.77; pooled HR 1.66, 95% CI 1.37–2.02). Incense burning was evaluated in a single study (OR 3.05, 95% CI 1.06–8.84), with wide confidence intervals. Two studies explored gene–environment interactions, suggesting possible variability in susceptibility, although statistical robustness was limited. **Conclusions**: Across multiple exposure categories, indoor air pollution was consistently associated with lung cancer risk, although the effect magnitude and precision varied between studies. Given the observational nature of the evidence and methodological heterogeneity, further prospective research with standardized exposure assessment is needed to clarify the strength and consistency of these associations.

## 1. Introduction

The first evidence connecting second-hand smoke with lung cancer came from Japan, from the Six-Prefecture Cohort Study by Takeshi Hirayama [1]. At the time, the idea that non-smokers could develop lung cancer simply by living with a smoker was unexpected. Hirayama’s work proved this association, revealing the carcinogenic nature of environmental tobacco smoke (ETS). It changed how passive exposure to second-hand smoke was viewed worldwide [2]. In the years that followed, several international studies confirmed these results across diverse populations, eventually shaping smoke-free policies in public and occupational settings [3,4,5].

Over half a century later, this message has evolved into a broader, more urgent concern: the air inside our homes, workplaces, and schools can itself be carcinogenic [6,7]. Today, scientists increasingly recognize that it is not only tobacco smoke that poses a risk. A variety of indoor pollutants—from the smoke produced by solid fuels to the vapors released during high-temperature cooking and everyday volatile organic compounds (VOCs)—are now linked to lung cancer [8,9,10]. This issue is particularly pressing in regions where biomass combustion and high-temperature cooking remain common, exposing women and other non-smoking groups to invisible yet persistent risks.

Globally, lung cancer is the leading cause of cancer-related mortality, claiming 1.8 million lives annually. Although smoking continues to account for most cases, 15–25% occurs in people who have never smoked [11]. For these individuals, chronic exposure to indoor air pollution represents one of the most plausible and often overlooked pathways. Indoors, carcinogenic compounds such as polycyclic aromatic hydrocarbons, aldehydes, nitrosamines, and fine particulate matter can accumulate to levels capable of inducing biological harm. More and more studies show that repeated inhalation of these pollutants triggers oxidative DNA injury, chromosomal instability, and dysregulation of genes involved in epithelial repair [12,13,14]. Taken together, these mechanisms explain how household or workplace exposures can, over time, contribute to lung carcinogenesis in people who have never smoked.

Although awareness of indoor air pollution has grown in recent years, its role in lung cancer has still received far less attention than smoking or outdoor air quality. For many years, research examined these exposures separately, even though real-world exposure is rarely isolated. Earlier reviews also faced significant limitations, such as different exposure measurement methods and rarely documented ventilation or housing conditions, which made comparisons difficult. Only a small number explored whether genetic susceptibility might intensify, alter, or decrease these risks. Together, these gaps have left the overall picture fragmented, contributing to persistent uncertainty about how indoor pollutants shape lung cancer risk across different settings.

This systematic review brings together the most recent evidence on how indoor air pollution contributes to lung cancer risk. Using clearly defined inclusion criteria and a consistent approach to quality and data extraction, we examined findings across major exposure domains: ETS, cooking oil fumes (COFs), use of solid fuels and biomass, and a small group of other domestic pollutants. Where studies allowed, we also considered how genetic susceptibility may modify these associations. Our goal was to assemble a coherent view of a field often fragmented by differing methods and contexts, and to highlight the gaps that still limit prevention efforts and the development of effective public health policy.

## 2. Materials and Methods

### 2.1. Study Design and Search Strategy

This systematic review was conducted in accordance with PRISMA 2020 recommendations, and the PRISMA checklist is included in the Supplementary Material Table S1. The review protocol was not prospectively registered. In October 2025, we performed a literature search across four databases: PubMed, Web of Science, Scopus, and the Cochrane Library. No date limits were applied at the search stage; however, as part of the predefined eligibility criteria, we restricted inclusion to studies published from 2015 onward to enhance the comparability of exposure definitions and multivariable adjustment practices in the contemporary indoor air pollution literature (e.g., clearer specification of household exposure metrics, ventilation-related variables, and more consistent reporting of adjusted effect estimates).

For PubMed, we used a predefined query combining MeSH terms and free-text keywords related to indoor air pollution and lung cancer as follows: (“Air Pollution, Indoor”[Mesh] OR “Household air pollution”[tiab] OR “indoor air pollution”[tiab] OR “cooking fumes”[tiab] OR biomass[tiab] OR “solid fuel”[tiab] OR coal[tiab] OR “wood smoke”[tiab] OR Incense[tiab] OR kerosene[tiab] OR “cooking oil fumes”[tiab] OR “particulate matter”[tiab] OR PM2.5[tiab] OR PM10[tiab] OR VOC[tiab] OR “volatile organic compounds”[tiab] OR “Tobacco Smoke Pollution”[Mesh] OR “environmental tobacco smoke”[tiab] OR “second-hand smoke”[tiab] OR “passive smoking”[tiab] OR “involuntary smoking”[tiab]) AND (“Lung Neoplasms”[Mesh] OR “lung cancer”[tiab] OR “pulmonary carcinoma”[tiab]) AND (“case-control”[tiab] OR “case control”[tiab] OR cohort[tiab] OR “nested case-control”[tiab] OR “case-cohort”[tiab]) NOT (radon[tiab] OR “in vitro”[tiab] OR animal[tiab] OR mice[tiab] OR rats[tiab] OR outdoor[tiab] OR traffic[tiab] OR “ambient air”[tiab]). The search yielded 565 records in PubMed.

The Web of Science Core Collection was queried in the Topic field (title, abstract, author keywords, keywords plus) using terms the terms “indoor air pollution”, “household air pollution”, “cooking fumes”, “solid fuel”, “wood smoke”, “biomass”, “coal”, “incense”, and “kerosene”, combined with “lung cancer”, “lung neoplasms”, “pulmonary carcinoma”, and “pulmonary neoplasms”. This yielded 268 records. The Scopus database was searched in the TITLE-ABS-KEY fields with the same exposure terms. The search was filtered to case–control or cohort designs, and excluded radon, in vitro, animal, and outdoor pollution studies. This search retrieved 344 records. The Cochrane Library was queried using equivalent exposure and outcome terms, and 10 records were returned; all were excluded during screening for not meeting the eligibility criteria. The full texts of potentially relevant studies were then assessed.

The search strategy was initially developed for PubMed and subsequently adapted to the field structure and indexing systems of the other databases, while preserving the same exposure and outcome concepts and Boolean logic to ensure methodological consistency across platforms.

### 2.2. Eligibility Criteria

The pre-established exclusion criteria were as follows:

1. Articles not published in English;
2. Publications dated before 2015;
3. Studies conducted exclusively on non-human models or unrelated laboratory settings;
4. Research addressing only radon, outdoor air pollution, or occupational exposure;
5. Studies reporting non-neoplastic respiratory outcomes;
6. Research on malignancies other than lung cancer;
7. Reviews, editorials, commentaries, letters, conference abstracts only, or duplicate records.

### 2.3. Study Selection and Data Extraction

All records identified through database searches were imported into a reference management system, and duplicate entries were removed prior to screening. Titles and abstracts were screened using Rayyan (Rayyan Systems Inc., Cambridge, MA, USA), a web-based systematic review management platform. Two reviewers independently screened titles and abstracts to assess eligibility. Full-text articles were evaluated against predefined exclusion criteria. Any discrepancies were resolved through discussion and, when necessary, consultation with a third reviewer to reach consensus. Full-text articles were excluded primarily due to exclusive focus on outdoor or occupational air pollution, non-neoplastic outcomes, or non-human study designs. Studies not meeting any of the exclusion criteria were considered eligible for inclusion if they evaluated indoor air pollution exposures in relation to lung cancer risk in human populations and reported effect estimates suitable for quantitative synthesis. The methodological quality and risk of bias of all included studies were assessed using the Newcastle–Ottawa Scale (NOS) for observational studies, with detailed scoring for each study provided in Supplementary Tables S2 and S3.

**Figure 1:**
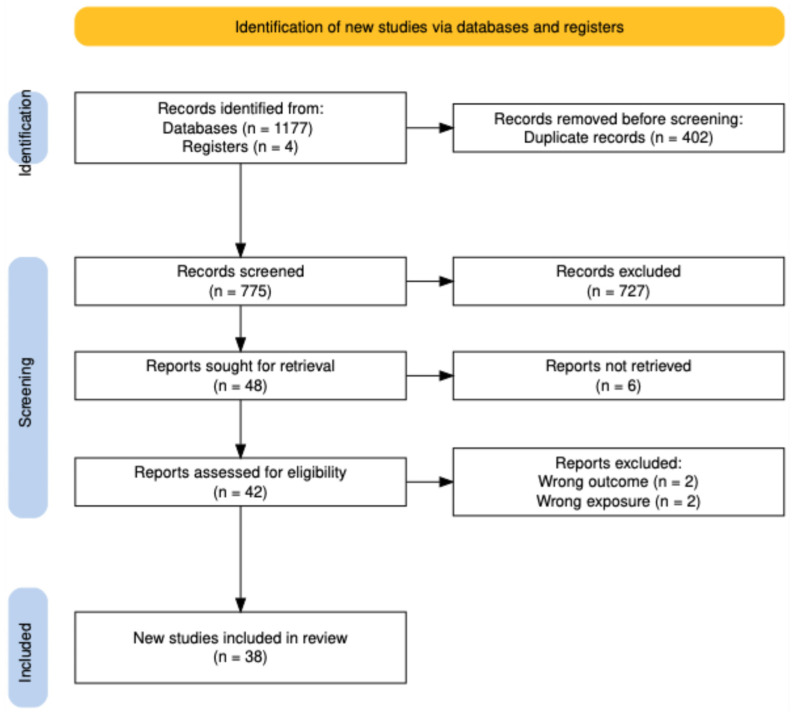
Prisma flowchart. Literature review.

The overall search process is summarized in the PRISMA flow diagram (Figure 1), which provides a step-by-step visual overview of how records were identified, screened, excluded, and, finally, included in the review. The main characteristics of the eligible studies are summarized in Table 1.

Data extraction was independently performed by two reviewers using a standardized data collection table. For each eligible study, we recorded study design, population characteristics, exposure definition and assessment method, outcome definition (primary lung cancer), covariates included in multivariable models, and maximally adjusted effect estimates with corresponding confidence intervals. When studies reported stratified analyses, particularly by smoking status, we extracted effect estimates among never-smokers to minimize confounding by active tobacco use and to better isolate the impact of indoor environmental exposures. When specific estimates for never-smokers were unavailable, the fully adjusted overall estimates were retained. Any discrepancies in extracted data were resolved through discussion and, when necessary, consultation with a third reviewer.

Exposure assessment methods varied across studies. Most investigations relied on structured or interviewer-administered questionnaires capturing exposure history, duration, and intensity. Several studies incorporated additional components such as genetic profiling, biomarker analyses, registry linkage, or exposure modeling approaches, while direct environmental monitoring was infrequently reported. This heterogeneity was documented during data extraction.

### 2.4. Statistical Analysis

Effect measures were transformed to the natural logarithm scale, and standard errors were derived from the reported 95% intervals. Pooled estimates were calculated separately for ORs and HRs using random-effects models (DerSimonian–Laird method). Between-study heterogeneity was evaluated using Cochran’s Q statistic and quantified with the I^2^ metric. The robustness of pooled results was explored through leave-one-out sensitivity analyses, in which each study was sequentially omitted to assess the stability of the summary estimate. Detailed results of the sensitivity are presented in the Supplementary Tables (Tables S4–S8). Given the limited number of studies within certain exposure categories and substantial heterogeneity in exposure assessment and adjustment strategies, additional modeling approaches were not undertaken. All statistical analyses were performed using MedCalc Statistical Software version 23.4.9 (MedCalc Software Ltd., Ostend, Belgium).

## 3. Results

We used Rayyan as a tool that facilitated paper screening. A total of 38 studies met the eligibility criteria and were included in the final synthesis, comprising 475,565 participants across Asia, Europe, North America, and Africa. The evidence base included both case–control and cohort study designs, between 2015 and 2025. Across studies, exposure assessment captured five distinct categories of indoor pollutants: ETS, COFs, solid fuels/biomass, incense exposure, and gene–environment interactions. The distribution of studies according to exposure category, study design, and reported lung cancer histology is summarized in Table 2. The following sections present the findings structured by exposure type.

### 3.1. Environmental Tobacco Smoke (ETS)

#### 3.1.1. Principal Findings and Interpretation

Across sixteen epidemiological studies, environmental tobacco smoke (ETS) was consistently associated with an increased risk of lung cancer. Excluding one descriptive hospital-based series from India, ten case–control studies contributed adjusted odds ratios for ETS exposure. The random-effects model yielded a pooled OR of 1.97 (95% CI 1.63–2.37), indicating that individuals exposed to ETS had approximately double the odds of developing lung cancer compared with unexposed participants. The heterogeneity was substantial (I^2^ = 77.9%), consistent with notable methodological variability across studies, including differences in exposure definitions, assessment periods, and population characteristics. Four cohort studies reported hazard ratios for ETS exposure. The random-effects model produced a pooled HR of 1.44 (95% CI 1.19–1.74), and unlike the OR-based evidence, heterogeneity across cohort studies was negligible (I^2^ = 0%), suggesting high internal consistency of ETS effects on lung cancer incidence across longitudinal designs. Leave-one-out sensitivity analyses confirmed the stability of the pooled estimates, with no single study altering the direction or statistical significance of the associations (Supplementary Tables S4 and S5).

**Figure 2:**
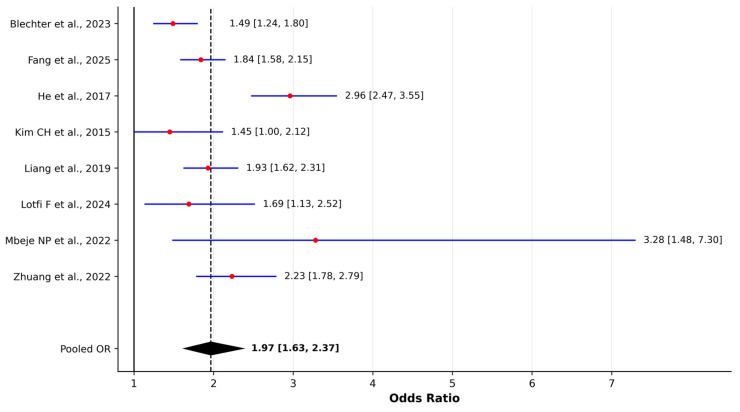
Forest plot of odds ratios (ORs) for environmental tobacco smoke (ETS) exposure and lung cancer risk in case–control studies [15,18,20,21,23,24,26,30]. Pooled estimates were calculated using a random-effects model (DerSimonian–Laird method). Red dots represent individual study effect estimates and blue lines indicate 95% confidence intervals. The diamond represents the pooled estimate with its 95% confidence interval. Between-study heterogeneity was assessed using Cochran’s Q test and I^2^ statistic.

**Figure 3:**
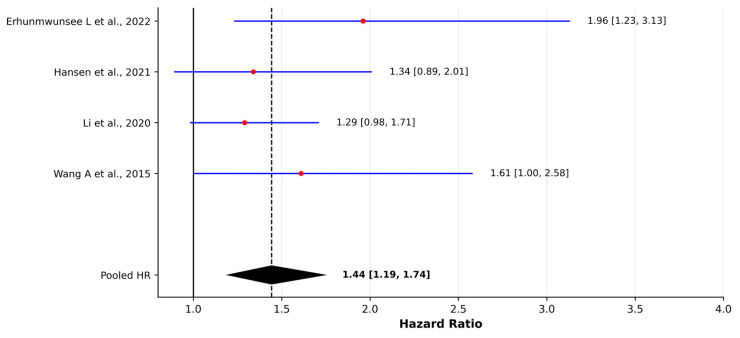
Forest plot of hazard ratios (HRs) for environmental tobacco smoke (ETS) exposure and lung cancer incidence in cohort studies [17,19,22,29]. Pooled estimates were calculated using a random-effects model (DerSimonian–Laird method). Red dots represent individual study effect estimates and blue lines indicate 95% confidence intervals. The diamond represents the pooled estimate with its 95% confidence interval. Between-study heterogeneity was assessed using Cochran’s Q test and I^2^ statistic.

We constructed separate forest plots for pooled odds ratios and hazard ratios to facilitate comparison across studies; these are presented in Figure 2 and Figure 3, respectively. Zhuang et al. [30] reported an overall ETS OR of 2.23 (95% CI 1.78–2.79), with sex-specific estimates of 2.22 in household-exposed women and 2.45 in workplace-exposed men. Similar magnitudes were observed by Liang D et al. [23], where both household and workplace ETS yielded ORs of 1.93 (95% CI 1.62–2.31) for home exposure and 1.93 (95% CI 1.53–2.42) for workplace exposure. He et al. [20] reported an adjusted OR of 2.96 (95% CI 2.47–3.55) for ETS in China, while Mbeje et al. [26] observed an aOR of 3.28 (95% CI 1.48–7.30) in a South African population. In the Iranian multicenter IROPICAN study, Lotfi F et al. reported an adjusted OR of 1.69 (95% CI 1.13–2.52) for exposure to second-hand tobacco smoke among never-smokers, further supporting a positive association between ETS and lung cancer risk.

Moderate risk associations were observed in prospective cohorts, such as those reported by Wang A et al. [29] with an HR 1.61 (95% CI 1.00–2.58) for 30 years or more of exposure, and Li J et al. (2020) [22], where ETS in never-smokers produced an HR 1.27 (0.98–1.71) after full adjustment, indicating a non-significant trend toward increased risk.

Overall, the direction and magnitude of associations were consistent with an increased lung cancer risk associated with environmental tobacco smoke exposure across diverse populations.

#### 3.1.2. Subgroup Trends and Effect Modifiers

When analyzed by sex, the risk was higher in women, particularly in household environments where exposure intensity and duration tend to be greater. Dose–response gradients were present in several studies, for example, Lotfii F et al. [24] reported an increasing OR with more prolonged daily exposure, reaching 2.29 (95% CI 1.20–4.37) for more than 3 h per day.

Genetic profile studies further contributed to heterogeneity. Soeroso et al. [27] shows an OR of 2.70 (95% CI 1.07–7.16) among the population with the CYP2A13 CT genotype, while Torres-Duran et al. [28] identified a striking synergy between the AAT SS genotype and 20 or more years of ETS exposure, resulting in an OR of 12.10 (95% CI 1.18–123.77); however, the wide confidence interval indicates limited precision and likely reflects sparse data due to the rarity of the genotype.

Despite these modifiers, the association remained directionally consistent across studies, including those adjusting for co-exposures such as cooking fumes or biomass fuels.

### 3.2. Cooking Oil Fumes (COFs)

#### 3.2.1. Principal Findings and Interpretation

**Figure 4:**
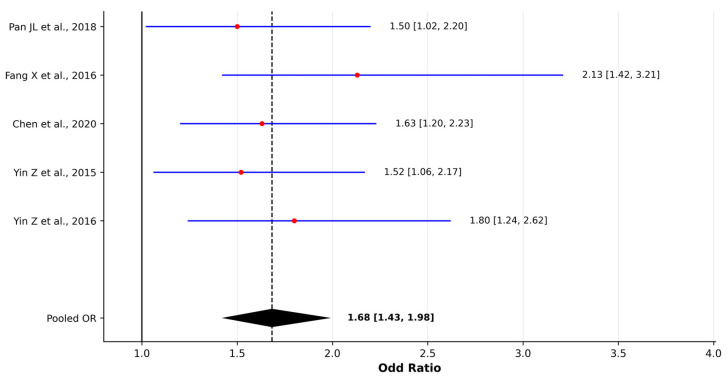
Forest plot of odds ratios (ORs) for cooking oil fume (COF) exposure and lung cancer risk in case–control studies [31,33,34,36,37]. Pooled estimates were calculated using a random-effects model (DerSimonian–Laird method). Red dots represent individual study effect estimates and blue lines indicate 95% confidence intervals. The diamond represents the pooled OR estimate with its 95% confidence interval. Between-study heterogeneity was assessed using Cochran’s Q test and I^2^ statistic.

Evidence from the seven eligible studies shows a consistent association between cooking oil fume (COF) exposure and increased lung cancer risk. Six case–control studies contributed effect estimates to the quantitative synthesis. Using a random-effects model, the pooled OR was 1.83 (95% CI 1.53–2.21), indicating a positive association across study settings. Between-study heterogeneity was low to moderate (I^2^ = 37.31%), likely reflecting differences in exposure assessment methods, cooking intensity metrics, and population characteristics. Leave-one-out sensitivity analyses did not materially alter the pooled estimate, and heterogeneity remained negligible upon sequential exclusion of individual studies (Supplementary Table S6), supporting the stability of the findings. The forest plot is displayed in Figure 4. One nationwide cohort study further examined occupational exposure to cooking fumes and reported an adjusted HR of 1.72 (95% CI 1.14–2.60), consistent in direction and magnitude with the pooled ORs from the case–control designs.

Hospital-based case–control studies in China reported some of the highest risks, with He F et al. [32] finding an OR of 2.63 (95% CI 1.91–3.61) for COF exposure in non-smoking women, reporting a positive association between COF exposure and lung cancer; however, as COFs were not the primary exposure and the degree of multivariable adjustment was insufficiently detailed, this estimate was excluded from the pooled analysis. Fang X et al. [33] reported an OR of 2.13 (95% CI 1.42–3.21) for cumulative COF exposure. In Taiwan, Chen et al. [34] showed a dose–response pattern with medium cumulative exposure to COFs being associated with an OR of 1.63 (95% CI 1.20–2.23), with higher risk at the upper end of exposure and partial risk reduction among long-term users of fume removers.

Occupational evidence pointed in the same direction, with a nationwide cohort of female school cooks (Jang J et al.) [35] observing a hazard ratio of 1.72 (95% CI 1.14–2.60) compared with clerks, suggesting that chronic occupational exposure to cooking fumes in institutional kitchens carries a risk of a similar magnitude to heavy domestic exposure.

Additional studies from the Liaoning region by Yin et al., 2015 [36] and Yin et al. 2016 [37] reported ORs of 1.52 and 1.80, respectively, again consistent with the broader evidence in this direction. Overall, the pattern across all eligible studies is coherent across different populations, designs, and strategies, converging on a harmful effect of COFs on lung cancer risk.

#### 3.2.2. Subgroup Trends and Effect Modifiers

Higher risks were generally seen in situations where people cooked for long periods, used high-heat methods, or worked in kitchens with limited ventilation. Studies that measured total cooking hours or captured specific habits such as frequent stir-frying or pan-frying showed clearer exposure–response trends than those using only broad frequency categories. In Chen et al., 2020 [34], both frequent pan-frying and greater lifetime cooking duration were associated with a higher risk, whereas regular use of a kitchen hood appeared to reduce the risk, with an adjusted OR close to 0.5.

Genetic susceptibility added further nuance, with several polymorphisms in miRNA-related genes and other loci showing higher combined risks when present together with COF exposure, but formal tests for interaction on additive or multiplicative scales were not always statistically significant.

Taken together, the main-effect signal for COFs remains stable across subgroups, with modifiers such as ventilation, cumulative exposure, and genetic background influencing the magnitude, rather than the presence, of excess risk.

### 3.3. Solid Fuels and Biomass

#### 3.3.1. Principal Findings and Interpretation

Based on the 12 eligible studies evaluating solid fuel and biomass exposure, the direction of association was generally consistent, with most investigations reporting elevated effect estimates: individuals who relied on coal, wood, or other smoke-generating fuels faced a higher risk of lung cancer than those using cleaner combustion sources. Most studies reported elevated adjusted odds ratios, while investigations conducted in high-exposure settings showed markedly stronger associations. Despite variability in exposure definitions and regional fuel characteristics, pooled analyses demonstrated a statistically significant association between solid fuel/biomass exposure and lung cancer risk. For case–control studies, the pooled OR was 2.26 (95% CI 1.36–3.77), with substantial between-study heterogeneity (I^2^ = 83%). In contrast, cohort-based estimates showed a pooled HR of 1.66 (95% CI 1.37–2.02) with no evidence of statistical heterogeneity (I^2^ = 0%). Leave-one-out sensitivity analyses confirmed the stability of both models (Supplementary Tables S7 and S8).

**Figure 5:**
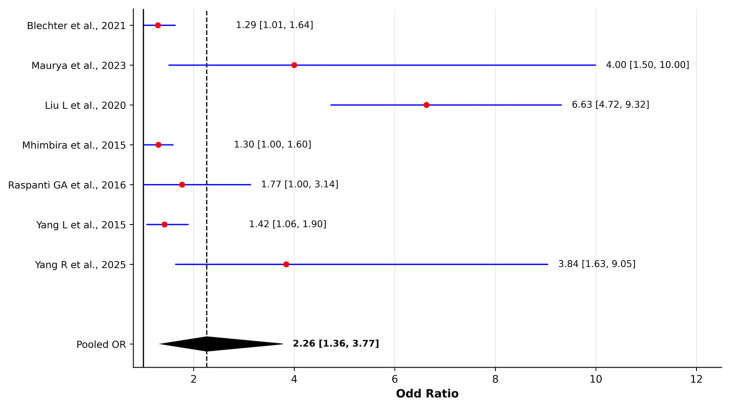
Forest plot of pooled odds ratios for the association between solid fuel/biomass exposure and lung cancer risk in case–control studies [38,39,45,46,47,48,49]. Pooled estimates were calculated using a random-effects model (DerSimonian–Laird method). Red dots represent individual study effect estimates and blue lines indicate 95% confidence intervals. The diamond represents the pooled effect estimate with 95% confidence interval. Between-study heterogeneity was assessed using Cochran’s Q test and I^2^ statistic.

**Figure 6:**
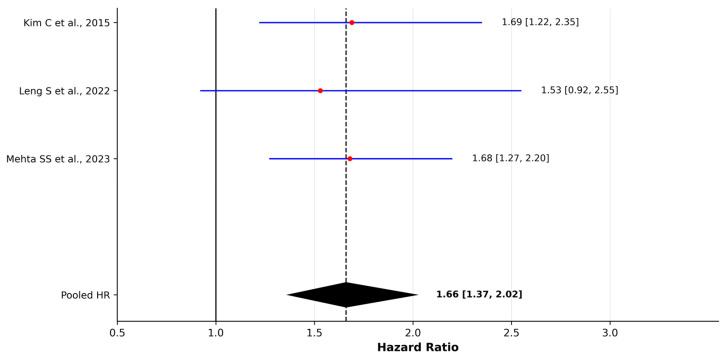
Forest plot of hazard ratios (HRs) for the association between solid fuel/biomass exposure and lung cancer risk in cohort studies [40,41,42]. Pooled estimates were calculated using a random-effects model (DerSimonian–Laird). Red dots represent individual study effect estimates and blue lines indicate 95% confidence intervals. The diamond represents the pooled effect estimate with 95% confidence interval. Between-study heterogeneity was assessed using Cochran’s Q test and I^2^ statistic.

In the Shanghai cohort, Kim C et al. [40] found an HR of 1.69 (95% CI 1.22–2.35), indicating that lung cancer risk increased when coal was used under poor kitchen ventilation, underscoring the combined relevance of fuel type and airflow conditions. Mehta SS et al. [42], using prospective data from the Sister Study, reported an HR of 1.68 (1.27–2.20), meaning that women engaging in indoor wood-burning for 30 days or more per year experienced a significantly increased incidence of lung cancer, highlighting that even intermittent domestic combustion in otherwise low-pollution environments may contribute to risk. To aid interpretation, we constructed separate forest plots for odds ratio and hazard ratio estimates, shown in Figure 5 and Figure 6, which outline the pattern of elevated risk across studies.

Two studies from China quantified exposure to smoky coal, historically highest risk fuel. Vermeulen R et al. [43] reported that the PAH25 exposure cluster was positively associated with lung cancer among never-smoking women (OR 2.21, 95% CI 1.67–2.87 per one standard deviation increase), after adjustment for age, second-hand smoke and socioeconomic status. Wong JYY et al. [44], with an OR of 33.40 (95% CI 13.07–85.34), identified extremely elevated odds ratios in households using high-emission deposits such as Laibin coal. However, the magnitude of this estimate likely reflects highly specific exposure conditions and regional fuel characteristics, and should be interpreted in the context of localized extreme emission profiles rather than generalized biomass exposure. Additional insights come from a recent matched case–control study by Yang R et al. [49], which reported an OR of 3.84 (95% CI 1.63–9.05), suggesting that prolonged indoor time may further modify the effective exposure dose beyond fuel type alone.

#### 3.3.2. Subgroup Trends and Effect Modifiers

Several factors influenced the strength of the observed associations for solid fuel and biomass exposure. Poor ventilation consistently amplified risk, as shown in Kim C et al. [40], where coal use in enclosed kitchens produced higher estimates than in well-ventilated settings. Fuel characteristics also played an important role, with studies from Xuanwei (Vermeulen et al. [43]; Wong JYY et al. [44]) demonstrating that smoky coal varieties carried a greater risk than smokeless coal, reflecting their higher emission profiles.

Behavioral context also further modified exposure. Yang R et al. found that individuals who spent more extended periods indoors accumulated higher effective doses, even when using similar fuels. Mehta SS et al. showed that repeated wood-burning in domestic environments may also contribute to risk over time [42].

Taken together, the evidence indicates a directionally consistent association between solid fuel combustion and lung cancer risk, with the magnitude of estimates influenced by the fuel’s emission characteristics, local ventilation conditions, and the time individuals spend indoors.

### 3.4. Incense Burning and Gene–Environment Interactions

#### 3.4.1. Incense Burning

Among the included studies, incense burning was identified as a distinct indoor exposure, particularly prevalent in certain cultural contexts. However, this exposure category was represented by a single case–control study (Chen et al. [50]). In that study, habitual incense use was associated with lung adenocarcinoma, with an adjusted OR of 3.05 (95% CI 1.06–8.84). A higher point estimate was observed among women (aOR 6.01), although the corresponding confidence interval was wide, indicating limited precision. Given that the evidence derives from a single study and exhibits imprecision, these findings should be interpreted cautiously and considered hypothesis-generating rather than confirmatory.

#### 3.4.2. Gene–Environment Interactions

Two studies evaluated whether genetic susceptibility modifies the association between indoor air pollution and lung cancer.

Ren Y et al. [51] examined the interaction between cooking oil fume exposure and TGFβ-1 polymorphisms in lung adenocarcinoma. In stratified analyses, individuals exposed to cooking oil fumes carrying the TGFβ-1 C509T TT genotype showed a reduced risk compared with the CC genotype (age-adjusted OR 0.36, 95% CI 0.15–0.88). Logistic regression models evaluating gene–environment interactions also yielded a reduced odds ratio for TT carriers exposed to COFs (age-adjusted OR 0.34, 95% CI 0.12–0.98), with a reported interaction *p* value of 0.046. However, after Bonferroni correction for multiple comparisons, these associations were no longer statistically significant. The findings, therefore, suggest possible effect modification, although statistical robustness was limited.

Wang G et al. [52] reported that longer daily cooking duration was associated with increased lung cancer incidence in a large prospective Chinese cohort. Participants who cooked for more than 2 h per day had an adjusted hazard ratio of 2.05 (95% CI 1.20–3.53) compared with non-cooking individuals. Joint effect analyses further indicated that carriers of the rs2395185 GT + TT genotype who cooked >2 h/day were associated with an adjusted hazard ratio of 2.48 (95% CI 1.03–5.97) compared with non-cooking individuals with the GG genotype, and similar elevated joint estimates were reported for rs3817963 variants. However, multiplicative interaction tests were not statistically significant. These findings suggest potential combined effects of prolonged cooking exposure and genetic background, although confirmation in larger cohorts is warranted.

Overall, although based on a limited number of studies, the findings suggest potential variability in risk estimates across genetic backgrounds.

## 4. Discussions

Indoor air pollution has long been overshadowed by the overwhelming impact of cigarette smoking on lung cancer epidemiology, but evidence accumulated over the past decade suggests that a proportion of lung cancer cases, especially among never-smokers, may be associated with household and environmental exposures [53]. The findings of this review support this perspective by demonstrating generally consistent associations across several exposure categories, although the magnitude and precision of estimates varied between studies.

The first and well-established determinant is environmental tobacco smoke (ETS). Most studies in our review reported an elevated risk, particularly in settings where exposure occurred over long periods in the home or indoor environment with inadequate ventilation. These results align with earlier pooled analyses [54] and continue to suggest that involuntary smoke exposure remains a relevant risk factor in populations where active smoking prevalence has declined. Sensitivity analyses using leave-one-out procedures did not materially alter the direction or statistical significance of pooled estimates, supporting the robustness of the association.

Cooking oil fumes were also associated with increased lung cancer risk in multiple studies, an important risk factor in regions where high-temperature frying, poor ventilation, and extended daily cooking durations are common. These findings are supported by studies demonstrating that hot oil aerosols contain aldehydes, PAHs, particulate matter, and other reactive species capable of inducing oxidative DNA damage and epithelial stress [55,56]. The fact that association patterns remain stable across domestic and occupational settings, as in large cohorts of school cooks, further underscores their robustness. Leave-one-out analyses confirmed the stability of pooled estimates, with minimal impact of any single study on overall effect size.

Solid fuel and biomass combustion were similarly associated with elevated lung cancer risk across most studies, particularly in poorly ventilated settings. When quantitatively synthesized, pooled estimates indicated a significantly increased risk. Substantial heterogeneity was observed in the case–control synthesis, likely reflecting differences in fuel types, combustion characteristics, exposure intensity, and regional contexts, whereas cohort-based estimates showed minimal statistical heterogeneity. Although heterogeneity was substantial in case–control studies, sensitivity analyses indicated that the association remained statistically significant even after excluding individual studies. In several high-emission settings, particularly coal-dependent regions, effect estimates were markedly elevated, suggesting that risk magnitude may depend strongly on exposure profile and environmental conditions.

Although supported by only a single eligible study, incense burning was identified as a distinct domestic exposure. The available evidence remains limited and does not allow firm conclusions. The observed association is biologically plausible, as incense combustion generates aromatic hydrocarbons and fine particulate matter that have been implicated in oxidative and inflammatory airway responses [57,58,59]. However, given the absence of replication and the wide confidence intervals reported, these findings should be interpreted cautiously and warrant further investigations.

Two studies addressed gene–environment interactions in the context of indoor air pollution and lung cancer. In one case–control study, associations between the TGFβ-1 polymorphisms and lung adenocarcinoma appeared to vary by cooking oil fume exposure. However, statistical significance did not persist after correction for multiple testing. In the prospective cohort study, prolonged daily cooking duration was independently associated with increased lung cancer incidence, and joint exposure–genotype analyses suggested higher risk estimates in selected polymorphism strata, although formal interaction tests were not statistically significant. Taken together, these findings indicate possible heterogeneity in susceptibility, but the evidence remains limited and statistically fragile. Larger, adequately powered studies with prespecified interaction evidence analyses are required before firm conclusions regarding gene–environment modification can be drawn.

### 4.1. Limitations

Despite the overall consistency of associations across exposure types, several methodological constraints limit the precision of the available estimates. Additionally, covariate adjustment strategies differed across studies. Age was consistently included in multivariable models, and most investigations adjusted for sex and active smoking status or cumulative pack-years. However, adjustment for socioeconomic indicators, body mass index, family history of lung cancer, occupational exposures, and comorbid lung disease was inconsistent. Only a subset of studies incorporated environmental co-exposures such as outdoor PM2.5, ventilation conditions, or concurrent indoor pollutants. This heterogeneity in confounder control limits the direct comparability of effect estimates and may partly explain the variability in magnitude across exposure categories. Residual confounding, therefore, cannot be excluded and should be considered when interpreting both pooled and narrative findings.

Most studies relied on self-reported exposure histories, introducing nondifferential misclassification that likely attenuates true risks. Objective exposure measures, such as airborne particulate monitoring or biomarker validation, were rarely used, and critical determinants, such as ventilation, stove efficiency, and housing structure, were inconsistently reported. Selection bias remains a concern in hospital-based case–control designs, while unmeasured or imperfectly captured co-exposures may also have influenced effect estimates.

An additional limitation relates to the geographical distribution of the included studies. A substantial proportion of investigations, particularly those evaluating cooking oil fumes and solid fuel/biomass exposure, were conducted in Asian populations. While this distribution reflects the higher prevalence of these exposure sources in specific regions, it may constrain the external validity of the findings in settings where household fuel types, cooking practices, ventilation standards, and background environmental exposures differ. Broader representation from underrepresented regions would strengthen the global applicability of future evidence.

Even so, the overall direction of associations was largely consistent across exposure categories, although the magnitude and precision of estimates varied substantially. In several studies, wide confidence intervals reflected limited sample size or rare exposure patterns, further contributing to statistical uncertainty. Associations observed for ETS, COFs, and solid fuel/biomass combustion are directionally consistent across multiple populations, although the magnitude varied. Evidence also suggests that combined or overlapping exposures may yield higher cumulative risk than single-pollutant models imply. Future studies should prioritize standardized exposure assessment, incorporate objective measurement strategies, and integrate behavioral, structural, and genetic modifiers to improve causal inference and support refined risk stratification for lung cancer prevention.

### 4.2. Clinical Relevance and Implications for Practice

The findings of this systematic review may have clinical relevance, particularly in the evaluation of lung cancer risk among individuals who fall outside traditional smoking-based risk frameworks. Lung cancer in never-smokers is increasingly recognized as a distinct clinical entity, characterized by specific epidemiological patterns and molecular features [60]. In this context, chronic indoor air pollution represents a potential exposure domain that may contribute to individualized risk assessment.

Although the associations observed across environmental tobacco smoke, cooking oil fumes, and solid fuel or biomass combustion were generally consistent, they derive predominantly from observational designs and varied in magnitude and precision. Therefore, indoor exposure histories should not be considered independent diagnostic criteria, but rather contextual factors that may complement established risk models.

Systematic inquiry into indoor exposure patterns, including household smoking, cooking practices, fuel type, ventilation conditions, and cumulative indoor time, may contribute to a more comprehensive contextual assessment of lung cancer risk, particularly among women and older adults, in whom non-tobacco-related lung cancer represents a meaningful proportion of cases [53,61]. Such exposure-aware evaluation may be informative when evaluating persistent respiratory symptoms, unexplained radiological abnormalities, or indeterminate pulmonary nodules in never-smokers, where pre-test probability of malignancy may otherwise be underestimated, and diagnostic delays can occur [62].

Overall, these findings support careful consideration of indoor exposure histories in clinical reasoning, while acknowledging that further prospective research is needed before formal integration into structured risk stratification frameworks.

## 5. Conclusions

Indoor air pollution was consistently associated with lung cancer risk across the studies included in this review. Adjusted effect estimates for the major exposure categories generally fell within a comparable range, although the magnitudes and precision varied substantially across studies.

From a public health perspective, these findings support continued investigation of indoor exposures as potentially modifiable risk factors. However, the current evidence remains predominantly observational, and further prospective research with standardized exposure assessment is required before integration into formal screening or risk stratification frameworks is considered.

In summary, the available evidence indicates that indoor air pollution represents a relevant exposure domain in lung cancer epidemiology. Greater awareness of household exposure patterns may contribute to improved contextual risk assessment, particularly among never-smokers and individuals outside traditional smoking-based risk categories.

## Figures and Tables

**Table 1:** Main characteristics of the eligible studies.

Author, Year	Country	Study Design	Exposure	Effect Estimate (95% CI)	N	Summary
Blechter et al., 2023 [15]	Taiwan	Case–control	ETS	OR 1.49 (1.24–1.80)	2048 (1024 cases; 1024 controls)	Female lifetime never-smokers; matched on age, sex, ethnicity; adjusted for age and 10 principal components; lung adenocarcinoma
Das et al., 2017 [16]	India	Hospital-based case series (prospective)	ETS	No comparative effect estimate reported	495	Descriptive; no OR/HR; limited generalizability
Erhumunneese L et al., 2022 [17]	USA	Prospective cohort	ETS	HR 1.96 (1.23–3.13)	37,650 (77 LC cases)	Never-smoking Black women; no matching; age, BMI, health insurance, education, income, ETS at home, PM2.5; incident lung cancer overall
Fang F et al., 2025 [18]	China	Population-based case–control	ETS	OR 1.84 (1.58–2.15)	10,890 (2871 cases; 8019 controls)	Adjusted for age, sex, income (10 y prior), education, county, family history of LC, smoking status, alcohol; mutual IAP adjustment; lung cancer overall
Hansen MS et al., 2021 [19]	Norway	Prospective cohort	ETS	HR 1.34 (0.89–2.01)	142,508 (1507 LC cases)	Never-smoking women, adjusted for age, education, and alcohol consumption; no significant association found; lung cancer overall
He F et al., 2017 [20]	China (Fujian)	Hospital case–control	ETS	OR 2.96 (2.47–3.55)	2206 (1096 cases; 1110 controls)	Adjusted for age, sex, education, occupation, marital status, BMI; primary lung cancer overall
Kim CH et al., 2015 [21]	USA (ILCCO pooled)	Pooled case–control	ETS	OR 1.45 (1.00–2.12)	3205 (170 cases; 3035 controls)	Female never-smokers; adjusted for age, sex, race/ethnicity; lung adenocarcinoma in situ; minimally invasive adenocarcinoma (AIS/MIA); heterogeneity reported
Li et al., 2020 [22]	China (Shanghai)	Prospective cohort	ETS	HR 1.29 (0.98–1.71)	23,415 (255 LC cases)	No significant association after full adjustment; adjusted for age, gender, education, income, smoking status, pack-years, BMI, alcohol, fat intake, fruit/vegetables; family history, lung cancer overall
Liang D et al., 2019 [23]	China (Hebei)	Matched case–control	ETS	OR 1.93 (1.62–2.31)	3248 (1086 cases; 2172 controls)	Never-smokers; conditional logistic regression; adjusted for sociodemographics, lifestyle factors, ETS, cooking, occupation, diet; lung cancer overall
Lotfi F et al., 2024 [24]	Iran	Multicenter case–control	ETS	OR 1.69 (1.13–2.52)	4104 (627 cases; 3477 controls)	Never-smokers; adjusted for age, sex, province, and SES; lung cancer overall
Masood et al., 2016 [25]	Pakistan	Case–control	ETS	Not reported separately	522 (252 cases; 270 controls)	No fully adjusted OR for ETS provided; lung cancer overall
Mbeje NP et al., 2022 [26]	South Africa	Case–control	ETS	aOR 3.28 (1.48–7.30)	234 (75 cases; 159 controls)	General population; adjusted for age, gender, race, marital status, education, alcohol, siblings, and history of lung cancer; lung cancer overall
Soeroso et al., 2021 [27]	Indonesia	Hospital case–control	ETS + CYP2A13 CT	OR 2.70 (1.07–7.16)	106 (53 cases; 53 controls)	Female never-smokers; no confounders specified (crude OR); lung cancer overall
Torres-Durán et al., 2015 [28]	Spain	Case–control	ETS + AAT SS genotype	OR 12.10 (1.18–123.77)	530 (5 SS cases out of 212; 318 controls)	Never-smokers; adjusted for age, gender, residential radon exposure; lung cancer overall
Wang A et al., 2015 [29]	USA (NIH centers)	Prospective cohort	ETS ≥ 30 years	HR 1.61 (1.00–2.58)	39,771	Never-smokers; adjusted for age, ethnicity, BMI, prior lung cancer, family cancer history, education, vitamin D, occupation, hormone therapy, lifestyle; lung cancer overall
Zhuang et al., 2022 [30]	China (Fujian)	Case–control	ETS	OR 2.23 (1.78–2.79)	1608 (623 cases; 985 controls)	Never-smokers; adjusted for age, nationality, education, marital status, BMI, alcohol, tea, lung disease history, occupation, family history of cancer; lung cancer overall
Pan JL et al., 2018 [31]	China (Shandong)	Case–control	COFs	OR 1.50 (1.02–2.20)	526 (261 cases; 265 controls)	Non-smoking women; adjusted for age, nationality, education, BMI, income, family history, passive smoking, occupation, soot; lung cancer overall
He F et al., 2017 [32]	China (Fujian)	Hospital case–control	COFs	OR 2.63 (1.91–3.61)	956 (477 cases; 479 controls)	Non-smoking women; Crude OR; lung cancer overall
Fang X et al., 2016 [33]	China (Shenyang)	Hospital case–control	COFs	OR 2.13 (1.42–3.21)	468 (224 cases; 244 controls)	Non-smoking women; age-adjusted; lung cancer overall
Chen et al., 2020 [34]	Taiwan	Case–control	Cooking time years (11–60 vs. ≤10)	OR 1.63 (1.20–2.23)	2604 (1302 cases; 1302 controls)	Non-smoking women; adjusted for age, education, family lung cancer, ETS exposure, HRT, oral contraceptive use, homemaker status, history of being a chef; mostly adenocarcinoma (84.6%)-1101
Jang J et al., 2025 [35]	Korea	Nationwide retrospective cohort	School kitchen COFs	HR 1.72 (1.14–2.60)	23,778	Female school cooks; adjusted for age, BMI, smoking, income, and CCI; PSM on age, BMI and CCI; lung cancer overall
Yin Z et al., 2015 [36]	China	Case–control	COFs	OR 1.52 (1.06–2.17)	568 (258 cases; 310 controls)	Non-smoking women; adjustment not specified for main COF model; adenocarcinoma (194), squamous cell carcinoma (34), other types (30)
Yin Z et al., 2016 [37]	China	Case–control	COFs	OR 1.80 (1.24–2.62)	534 (268 cases; 266 controls)	Non-smokers; adjustment not specified; 197 adenocarcinoma, 44 squamous cell, 27 other lung cancer types
Blechter et al., 2021 [38]	China (Shanghai, Shenyang)	GWAS-based case–control	Household coal use (ever vs. never)	OR 1.29 (1.01–1.64)	2297 (1183 cases; 1114 controls)	Never-smoking women; adjusted for age and study; adenocarcinoma
Maurya et al., 2023 [39]	India (W. Maharashtra)	Case–control	Smoke-generating fuel	OR 4.00 (1.5–10.0)	136 (68 cases; 68 controls)	Crude OR; hospital-based sample; small sample size
Kim C et al., 2015 [40]	China (Shanghai)	Prospective cohort	Coal use + poor ventilation	HR 1.69 (1.22–2.35)	71,320	Never-smoking women; adjusted for age, education, income, ETS, history of lung disease, occupation; incident lung cancer overall
Leng S et al., 2022 [41]	USA (New Mexico)	Prospective cohort	Wood smoke exposure (≥1 year)	HR 1.53 (0.92–2.55)	2372	Ever-smokers; adjusted for age, smoking status and PY, sex, ethnicity; non-significant association; incident lung cancer overall
Mehta SS et al., 2023 [42]	USA (Sister Study)	Prospective cohort	Indoor wood-burning ≥30 days/year	HR 1.68 (1.27–2.20)	50,226 women	Adjusted for race, ethnicity, education, residential status, smoking, PY, ETS, marital status, income; incident primary lung cancer;
Vermeulen R et al., 2019 [43]	China (Xuanwei & Fuyuan)	Case–control	Smoky coal exposure (PAH25 per 1 SD)	OR 2.21 (1.67–2.87)	1500 (1015 cases; 485 controls)	Never-smoking women; adjusted for age, ETS, SES (food sufficiency); overall lung cancer
Wong JYY et al., 2019 [44]	China (Xuanwei & Fuyuan)	Case–control	Smoky coal exposure (by geologic deposit)	OR 33.40 (13.07–85.34)	1524 (1031 cases; 493 controls)	Female never-smokers; adjusted for age and food sufficiency before marriage or age; extreme effect estimate
Liu L et al., 2020 [45]	China (Yunnan)	Case–control	Smoky coal exposure (high vs. low exposure)	OR 6.63 (4.72–9.32)	3000 (684 cases; 2316 controls)	Adjusted for age, sex, heating method, occupation, diet, alcohol, lung disease history, family history, education; overall lung cancer
Mhimbira et al., 2015 [46]	Asia (FLC Cons.)	Pooled case–control	Coal use (ever vs. never)	OR 1.30 (1.00–1.60)	3080 (1731 cases; 1349 controls)	Never-smoking women; adjusted for age and study; lung cancer overall
Raspanti GA et al., 2016 [47]	Nepal	Hospital-based case–control	Biomass burning	OR 1.77 (1.00–3.14)	1212 (606 cases; 606 controls)	Adjusted for age, sex, ethnicity, tobacco use, ETS, SES, tobacco status + PY; lung cancer overall
Yang L et al., 2015 [48]	China	Case–control	Solid fuel use; Southern China	OR 1.42 (1.06–1.90)	2112 (1056 cases; 1056 controls)	Adjusted for age, sex, BMI, education; lung cancer overall
Yang R et al., 2025 [49]	China (Beijing)	Matched case–control	HAP + indoor time spent (>23 h/day for ≥10 years)	OR 3.84 (1.63–9.05)	286 (143 cases; 143 controls)	Female never-smokers; adjusted for age, sex, BMI, diet; lung adenocarcinoma
Chen et al., 2022 [50]	Taiwan	Case–control	Incense burning	OR 3.05 (1.06–8.84)	79 (39 cases; 40 controls)	Age-adjusted; wide CI; small sample size; lung adenocarcinoma
Ren Y et al., 2015 [51]	China (Liaoning)	Case–control	Gene–COF interaction	OR 0.34 (0.12–0.98)	585 (272 cases; 313 controls)	Age-adjusted logistic regression only; interaction not significant after Bonferroni correction; lung adenocarcinoma
Wang G et al., 2019 [52]	China (Dongfeng–Tongji)	Prospective cohort	Gene–cooking time interaction	HR 2.48 (1.03–5.97)	33868 (5178 genotyped)	Adjusted for age, gender, marital status, education, smoking, alcohol, exercise, BMI, family history of cancer, benign lung disease, housing characteristics, and occupational factors; incident lung cancer overall

Abbreviations: AAT, alpha-1 antitrypsin; AIS, adenocarcinoma in situ; MIA, minimally invasive adenocarcinoma; CCI, Charlson Comorbidity Index; CI, confidence interval; COF, cooking oil fume; ETS, environmental tobacco smoke; GWAS, genome-wide association study; HR, hazard ratio; ILCCO, International Lung Cancer Consortium; OR, odds ratio; PAH, polycyclic aromatic hydrocarbons; PM2.5, particulate matter ≤ 2.5 µm; PSM, propensity score matching; PY, pack-years; SD, standard deviation; SES, socioeconomic status.

**Table 2:** Distribution of included studies by indoor pollutant category, study design, and reported cancer histology.

Exposure Category	No. of Studies (*n*)	Study Design	Reported Histology
ETS	16	Case–control (*n* = 12); Cohort (*n* = 4)	Primary lung cancer (overall); adenocarcinoma
COF	7	Case–control (*n* = 6); Cohort (*n* = 1)	Primary lung cancer (overall); adenocarcinoma, squamous cell carcinoma, other
Solid fuels/biomass	12	Case–control (*n* = 9); Cohort (*n* = 3)	Primary lung cancer (overall); adenocarcinoma
Incense	1	Case–control (*n* = 1)	Adenocarcinoma
Gene–environment interactions	2	Case–control (*n* = 1); Cohort (*n* = 1)	Primary lung cancer (overall); adenocarcinoma

All outcomes refer to primary lung cancer. Where histology was reported, subtypes are specified.

## Supplementary Materials

The following supporting information can be downloaded at: https://www.mdpi.com/article/10.3390/jcm15051854/s1, File S1: Research protocol; Table S1: PRISMA Checklist [63]; Table S2: Newcastle–Ottawa Assessment Scale for case–control studies [15,18,20,21,23,24,26,27,28,30,31,32,33,34,36,37,38,39,43,44,45,46,47,48,49,50,51]; Table S3: Newcastle–Ottawa Assessment Scale for cohort studies [17,19,22,29,35,40,41,42,52]; Table S4: Leave-one-out for pooled ORs of ETS case-control studies [15,18,20,21,23,24,26,30]; Table S5: Leave-one-out for pooled HRs of ETS cohort studies [17,19,22,29]; Table S6: Leave-one-out for pooled ORs of COF case-control studies [31,33,34,36,37]; Table S7: Leave-one-out for pooled ORs of solid fuels/biomass case-control studies [38,39,45,46,47,48,49]; Table S8: Leave-one-out for pooled HRs of solid fuels/biomass cohort studies [40,41,42].
