# Supplementary material for: Indoor Air Pollution and Lung Cancer Risk—A Systematic Review and Meta-Analysis
Source: J Clin Med. 2026 Feb 28;15(5):1854. doi: 10.3390/jcm15051854 (PMC12986033; doi:10.3390/jcm15051854)
Supplement: Supplementary file 1 [file jcm-15-01854-s001.zip › jcm-4133079-supplementary.pdf]

Table S1. PRISMA Checklist

Section and Topic	Item #	Checklist item	Location where item is reported
**TITLE**			
Title	1	Identify the report as a systematic review.	Title page – p. 1
**ABSTRACT**			
Abstract	2	See the PRISMA 2020 for Abstracts checklist.	Abstract – p. 1
**INTRODUCTION**			
Rationale	3	Describe the rationale for the review in the context of existing knowledge.	Introduction, paragraph 1-4 – pp. 2-3
Objectives	4	Provide an explicit statement of the objective(s) or question(s) the review addresses.	Introduction, final paragraph – p. 3
**METHODS**			
Eligibility criteria	5	Specify the inclusion and exclusion criteria for the review and how studies were grouped for the syntheses.	Materials and Methods – Exclusion criteria subsection - p. 7
Information sources	6	Specify all databases, registers, websites, organizations, reference lists and other sources searched or consulted to identify studies. Specify the date when each source was last searched or consulted.	Materials and Methods – Database search description – pp. 3-4
Search strategy	7	Present the full search strategies for all databases, registers and websites, including any filters and limits used.	Materials and Methods – Database search description – pp. 3-5
Selection process	8	Specify the methods used to decide whether a study met the inclusion criteria of the review, including how many reviewers screened each record and each report retrieved, whether they worked independently, and if applicable, details of automation tools used in the process.	Materials and Methods – Study selection paragraph – p.5
Data collection process	9	Specify the methods used to collect data from reports, including how many reviewers collected data from each report, whether they worked independently, any processes for obtaining or confirming data from study investigators, and if applicable, details of automation tools used in the process.	Materials and Methods – Effect estimate extraction paragraph – pp. 5-6
Data items	10a	List and define all outcomes for which data were sought. Specify whether all results that were compatible with each outcome domain in each study were sought (e.g., for all measures, time points, and analyses), and if not, the methods used to decide which results to collect.	Materials and Methods – Data extraction paragraph – pp. 5-6
	10b	List and define all other variables for which data were sought (e.g., participant and intervention characteristics, funding sources). Describe any assumptions made about any missing or unclear information.	Materials and Methods – Data extraction

Section and Topic	Item #	Checklist item	Location where item is reported
			paragraph – pp. 5-6
Study risk of bias assessment	11	Specify the methods used to assess risk of bias in the included studies, including details of the tool(s) used, how many reviewers assessed each study and whether they worked independently, and if applicable, details of automation tools used in the process.	Materials and Methods – Data extraction and quality assessment – p. 5
Effect measures	12	Specify for each outcome the effect measure(s) (e.g., risk ratio, mean difference) used in the synthesis or presentation of results.	Materials and Methods – Statistical analysis – p. 6
Synthesis methods	13a	Describe the processes used to decide which studies were eligible for each synthesis (e.g., tabulating the study intervention characteristics and comparing against the planned groups for each synthesis (item #5)).	Materials and Methods – Data extraction and synthesis – pp. 5-6
	13b	Describe any methods required to prepare the data for presentation or synthesis, such as handling of missing summary statistics, or data conversions.	Materials and Methods – Statistical analysis – p. 6
	13c	Describe any methods used to tabulate or visually display results of individual studies and syntheses.	Results – Table 1 and Figures 2-6 – pp. 8-16
	13d	Describe any methods used to synthesize results and provide a rationale for the choice(s). If meta-analysis was performed, describe the model(s), method(s) to identify the presence and extent of statistical heterogeneity, and software package(s) used.	Materials and Methods – Statistical analysis – p.6
	13e	Describe any methods used to explore possible causes of heterogeneity among study results (e.g., subgroup analysis and meta-regression).	Materials and Methods – Statistical analysis – p. 6
	13f	Describe any sensitivity analyses conducted to assess robustness of the synthesized results.	Materials and Methods – Statistical analysis – p. 6
Reporting bias assessment	14	Describe any methods used to assess risk of bias due to missing results in a synthesis (arising from reporting biases).	Not assessed
Certainty assessment	15	Describe any methods used to assess certainty (or confidence) in the body of evidence for an outcome.	Not assessed
**RESULTS**			
Study selection	16a	Describe the results of the search and selection process, from the number of records identified in the search to the number of studies included in the review, ideally using a flow diagram.	Results and Figure 1 – p. 8
	16b	Cite studies that might appear to meet the inclusion criteria, but which were excluded, and explain why they were excluded.	Results – PRISMA flow diagram (Figure 1) – p.

Section and Topic	Item #	Checklist item	Location where item is reported
			8
Study characteristics	17	Cite each included study and present its characteristics.	Results – Table 1 – pp. 9-11
Risk of bias in studies	18	Present assessments of risk of bias for each included study.	Supplementary Tables S2-S3
Results of individual studies	19	For all outcomes, present, for each study: (a) summary statistics for each group (where appropriate) and (b) an effect estimate and its precision (e.g., confidence/credible interval), ideally using structured tables or plots.	Results – Figures 2-6 – pp. 9-16
Results of syntheses	20a	For each synthesis, briefly summarize the characteristics and risk of bias among contributing studies.	Results – exposure specific sections and Table 1 – pp. 9-15
	20b	Present results of all statistical syntheses conducted. If meta-analysis was done, present for each the summary estimate and its precision (e.g., confidence/credible interval) and measures of statistical heterogeneity. If comparing groups, describe the direction of the effect.	Results – data analysis sections – pp. 10-15
	20c	Present results of all investigations of possible causes of heterogeneity among study results.	Results – data analysis sections
	20d	Present results of all sensitivity analyses conducted to assess the robustness of the synthesized results.	Results – sensitivity analyses – pp. 10-15
Reporting biases	21	Present assessments of risk of bias due to missing results (arising from reporting biases) for each synthesis assessed.	Not assessed
Certainty of evidence	22	Present assessments of certainty (or confidence) in the body of evidence for each outcome assessed.	Not assessed
**DISCUSSION**			
Discussion	23a	Provide a general interpretation of the results in the context of other evidence.	Discussion – pp. 17-20
	23b	Discuss any limitations of the evidence included in the review.	Discussion – limitations – pp. 18-19
	23c	Discuss any limitations of the review processes used.	Discussion – pp. 19
	23d	Discuss implications of the results for practice, policy, and future research.	Discussion – implications – pp. 20-21
**OTHER INFORMATION**			
Registration and protocol	24a	Provide registration information for the review, including register name and registration number, or state that the review was not registered.	Materials and methods – first paragraph – p. 3
	24b	Indicate where the review protocol can be accessed, or state that a protocol was not prepared.	Not applicable

Section and Topic	Item #	Checklist item	Location where item is reported
	24c	Describe and explain any amendments to information provided at registration or in the protocol.	Not applicable
Support	25	Describe sources of financial or non-financial support for the review, and the role of the funders or sponsors in the review.	Funding – p. 22
Competing interests	26	Declare any competing interests of review authors.	Conflict of interest section – p. 22
Availability of data, code and other materials	27	Report which of the following are publicly available and where they can be found: template data collection forms; data extracted from included studies; data used for all analyses; analytic code; any other materials used in the review.	Data availability statement – p. 22

From: Page MJ, McKenzie JE, Bossuyt PM, Boutron I, Hoffmann TC, Mulrow CD, et al. The PRISMA 2020 statement: an updated guideline for reporting systematic reviews. BMJ 2021;372:n71. doi: 10.1136/bmj.n71. This work is licensed under CC BY 4.0. To view a copy of this license, visit https://creativecommons.org/licenses/by/4.0/

Table S2. Newcastle–Ottawa Assessment Scale for case–control studies [15,18,20,21,23,24,26,27,28,30,31-34,36,37–39,43–51].

Study	Selection (4)				Comparability (2)		Outcome (3)			Total score	Risk of Bias
Blechter et al., 2023	★	★	★	★	★	★	★	★	☆	8	Low
Fang F et al., 2025	★	★	★	★	★	★	★	★	☆	7	Low
He F et al., 2017	★	★	☆	★	★	★	★	★	☆	7	Low
Kim CH et al., 2015	★	★	★	★	★	★	★	★	☆	8	Low
Liang D et al., 2019	★	★	★	★	★	★	★	★	☆	8	Low
Lotfi F et al., 2024	★	★	★	★	★	★	★	★	★	9	Low
Mbeje NP et al., 2022	★	★	★	★	★	★	★	★	☆	8	Low
Soeroso et al., 2021	★	★	★	★	★	☆	★	★	☆	7	Low
Torres-Durán et al., 2015	★	★	★	★	★	★	★	★	☆	8	Low
Zhuang et al., 2022	★	★	★	★	★	★	★	★	☆	8	Low
Pan JL et al., 2018	★	☆	★	★	★	★	★	★	☆	7	Low
He F et al., 2017	★	★	★	★	★	★	★	★	☆	8	Low
Fang X et al., 2016	★	★	★	★	★	☆	★	★	☆	7	Low
Chen et al., 2020	★	★	★	★	★	★	★	★	★	9	Low
Yin Z et al., 2015	★	★	★	★	★	★	★	★	☆	8	Low
Yin Z et al., 2016	★	★	★	★	★	☆	★	★	☆	7	Low
Blechter et al., 2021	★	★	★	★	★	★	★	★	☆	8	Low
Maurya et al., 2023	★	☆	☆	★	★	☆	★	★	☆	5	Moderate
Vermeulen R et al., 2019	★	★	★	★	★	★	★	★	★	9	Low
Wong JYY et al., 2019	★	★	★	★	★	★	★	★	★	9	Low
Liu L et al., 2020	★	★	★	★	★	★	★	★	☆	8	Low
Mhimbira et al., 2015	★	★	★	★	★	★	★	★	☆	8	Low
Raspanti GA et al., 2016	★	★	★	★	★	★	★	★	☆	8	Low
Yang L et al., 2015	★	★	★	★	★	★	★	★	☆	8	Low
Yang R et al., 2025	★	☆	★	★	★	★	★	★	☆	7	Low
Chen et al., 2022	★	★	★	★	★	★	★	★	★	9	Low
Ren Y et al., 2015	★	★	★	★	★	☆	★	★	☆	7	Low

Table S3. Newcastle–Ottawa Assessment Scale for cohort studies [17,19,22,29,35,40–42,52].

Study	Selection (4)				Comparability (2)		Outcome (3)			Total score	Risk of Bias
Erhumunneese L et al., 2022	★	★	★	★	★	☆	★	★	★	8	Low
Hansen MS et al., 2021	★	★	★	★	★	☆	★	★	★	8	Low
Li J et al., 2020	★	★	★	★	★	★	★	★	☆	8	Low
Wang A et al., 2015	★	★	★	★	★	★	★	★	★	9	Low
Kim C et al., 2015	★	★	★	★	★	★	★	★	★	9	Low
Leng S et al., 2022	★	★	★	★	★	☆	★	★	★	8	Low
Mehta SS et al., 2023	★	★	★	★	★	★	★	★	★	9	Low
Wang G et al., 2019	★	★	★	★	★	★	★	☆	★	8	Low
Jang J et al., 2025	★	★	★	★	★	★	★	★	★	9	Low

Table S4. Leave-one-out for pooled ORs of ETS case-control studies [15,18,20,21,23,24,26,30].

Omitted Study	Pooled OR	95% CI	I^2 (%)	P value
Blechter et al., 2023	2.07	[1.71, 2.50]	74.8%	0.0000
Fang et al., 2025	1.99	[1.58, 2.51]	82.1%	0.0000
He et al., 2017	1.82	[1.59, 2.07]	48.4%	0.0000
Kim CH et al., 2015	2.04	[1.67, 2.48]	81.2%	0.0000
Liang et al., 2019	1.97	[1.57, 2.48]	82.6%	0.0000
Lotfi F et al., 2024	2.00	[1.63, 2.45]	82.4%	0.0000
Mbeje et al., 2022	1.92	[1.59, 2.33]	81.9%	0.0000
Zhuang et al., 2022	1.93	[1.55, 2.39]	82.0%	0.0000

Table S5. Leave-one-out for pooled HRs of ETS cohort studies [17,19,22,29].

Omitted Study	Pooled HR	95% CI	I^2 (%)	P value
Erhunmwunsee L et al., 2022	1.36	[1.11, 1.67]	0.0%	0.0037
Hansen et al., 2021	1.50	[1.17, 1.92]	18.2%	0.0012
Li et al., 2020	1.59	[1.23, 2.06]	0.0%	0.0004
Wang A et al., 2015	1.43	[1.13, 1.79]	15.3%	0.0024

Table S6. Leave-one-out for pooled ORs of COF case-control studies [31,33,34,36,37].

Omitted Study	Pooled OR	95% CI	I^2 (%)	P value
Pan JL et al., 2018	1.72	[1.44, 2.06]	0.0%	0.0000
Fang X et al., 2016	1.61	[1.35, 1.92]	0.0%	0.0000
Chen et al., 2020	1.70	[1.41, 2.06]	0.0%	0.0000
Yin Z et al., 2015	1.73	[1.44, 2.07]	0.0%	0.0000
Yin Z et al., 2016	1.66	[1.38, 1.98]	0.0%	0.0000

Table S7. Leave-one-out for pooled ORs of solid fuels/biomass case-control studies [38,39,45–49].

Omitted Study	Pooled OR	95% CI	I^2 (%)	P value
Blechter et al., 2021	2.54	[1.35, 4.79]	92.9%	0.0038
Maurya et al., 2023	2.11	[1.23, 3.63]	93.4%	0.0067
Liu L et al., 2020	1.57	[1.23, 2.01]	56.4%	0.0003
Mhimbira et al., 2015	2.54	[1.35, 4.80]	92.9%	0.0041
Raspanti GA et al., 2016	2.36	[1.33, 4.19]	93.7%	0.0033
Yang L et al., 2015	2.49	[1.33, 4.67]	93.5%	0.0043
Yang R et al., 2025	2.11	[1.23, 3.64]	93.4%	0.0070

Table S8. Leave-one-out for pooled HRs of solid fuels/biomass cohort studies [40–42].

Omitted Study	Pooled HR	95% CI	I^2 (%)	P value
Kim C et al., 2015	1.65	[1.29, 2.10]	0.0%	0.0001
Leng S et al., 2022	1.68	[1.36, 2.08]	0.0%	0.0000
Mehta SS et al., 2023	1.64	[1.25, 2.16]	0.0%	0.0004

File S1. Research protocol

Indoor Air Pollution and Lung Cancer Risk – A Systematic Review and Meta-Analysis

Systematic review – research protocol

1. Review title

Within Four Walls: Indoor Air Pollution and Lung Cancer Risk – A systematic review (can change with time)

2. Health problems and exposure

The review addresses primary lung cancer in adults in relation to indoor/household air pollution exposure: environmental tobacco smoke, cooking oil fumes, solid fuel/biomass combustion, and related household combustion sources.

3. Objectives

- To estimate the association between indoor air pollution exposures and primary lung cancer risk in human populations.
- To pool maximally adjusted ORs and HRs where studies are sufficiently comparable.
- To explore heterogeneity by exposure type, study design.

4. Eligibility criteria

Types of studies

- Inclusion: Observational epidemiologic studies (case–control and cohort).
- Exclusion: RCTs, ecological designs, case reports/series without a comparison group, reviews, editorials, letters, and conference abstracts only.

Participants

- Human participants of any sex and adult age from any geographic region.
- Studies exclusively on animals or in vitro models are excluded.

Exposures

- Included: Indoor/household exposures such as environmental tobacco smoke, cooking fumes/oil fumes, solid fuels (coal, wood, and biomass), kerosene, incense, and other clearly indoor combustion sources or indoor particulate/volatile pollutants.
- Excluded: Studies focusing only on radon, outdoor/ambient air pollution, traffic-related, or purely occupational exposures without a household/indoor component.

Outcomes

- Included: Primary lung cancer incidence or mortality, overall or by histological subtype (e.g., adenocarcinoma).
- Excluded: Non-neoplastic respiratory outcomes and malignancies other than lung cancer.

Setting and timeframe

- Any country and setting (community or hospital-based), provided the exposure is indoor/household.
- Publication year: 2015 onwards (to improve comparability of exposure definitions and multivariable adjustment).
- Language: English

5. Information sources

- Databases: PubMed, Web of Science Core Collection, Scopus, and Cochrane Library.
- Date of last search: October 2025.
- Year restriction applied at the eligibility stage (≥2015).

6. Search strategy

A predefined PubMed strategy combined MeSH and free-text terms for indoor air pollution and lung neoplasms, along with observational study design filters, and excluded radon, animal, in vitro, and outdoor pollution terms. This strategy was translated into Web of Science (Topic), Scopus (TITLE-ABS-KEY), and Cochrane, using equivalent concepts and Boolean logic.

PubMed string: ("Air Pollution, Indoor"[Mesh] OR "Household air pollution"[tiab] OR "indoor air pollution"[tiab] OR "cooking fumes"[tiab] OR biomass[tiab] OR "solid fuel"[tiab] OR coal[tiab] OR "wood smoke"[tiab] OR Incense [tiab] OR kerosene[tiab] OR "cooking oil fumes"[tiab] OR "particulate matter"[tiab] OR PM2.5[tiab] OR PM10[tiab] OR VOC[tiab] OR "volatile organic compounds"[tiab] OR "Tobacco Smoke Pollution"[Mesh] OR "environmental tobacco smoke"[tiab] OR "second-hand smoke"[tiab] OR "passive smoking"[tiab] OR "involuntary smoking"[tiab]) AND ("Lung Neoplasms"[Mesh] OR "lung cancer"[tiab] OR "pulmonary carcinoma"[tiab]) AND ("case-control"[tiab] OR "case control"[tiab] OR cohort[tiab] OR "nested case-control"[tiab] OR "case-cohort"[tiab]) NOT (radon[tiab] OR "in vitro"[tiab] OR animal[tiab] OR mice[tiab] OR rats[tiab] OR outdoor[tiab] OR traffic[tiab] OR "ambient air"[tiab]).

7. Study records: data management and selection

- All records were imported into a reference manager; duplicates were removed prior to screening.
- Two reviewers independently screened titles/abstracts and then full texts against the eligibility criteria; disagreements were resolved by discussion or a third reviewer.
- Screening flow: databases 1,177 + registers 4; duplicates 402; screened 775; excluded 727; reports sought 48; not retrieved 6; full texts assessed 42; excluded for wrong outcome 2 and wrong exposure 2; included 38.

8. Data items and extraction

From each study, we planned to extract: study design, country/setting, sample size, population characteristics (age, sex, and smoking status), exposure definition and assessment, outcome definition (primary lung cancer and histology), confounders used in multivariable models, and maximally adjusted effect estimates (ORs and HRs) with 95% CIs.

Data extraction was performed using a standardized table by one reviewer and checked independently by a second reviewer; discrepancies were resolved by consensus. When studies provided subgroup estimates by smoking status, we preferentially extracted estimates for never-smokers.

9. Risk of bias (quality) assessment

Risk of bias in individual studies was assessed using the Newcastle–Ottawa Scale (NOS) for observational studies, covering selection, comparability, and outcome/exposure domains. NOS judgments were used descriptively and in sensitivity analyses.

10. Outcomes and prioritization

Primary outcome:

- Association (OR or HR) between indoor air pollution exposure and primary lung cancer incidence.

Secondary aspects:

- Histologic subtype-specific associations when specified (e.g., adenocarcinoma).
- Gene–environment interaction results were reported and treated descriptively.

11. Data synthesis

- Effect measures: maximally adjusted ORs and HRs.

- For meta-analysis, log-transformed ORs/HRs and standard errors were calculated from reported effect estimates and 95% CIs.
- When ≥3 sufficiently homogeneous studies were available for a given exposure and effect measure, random-effects models were used to obtain pooled estimates, with heterogeneity quantified using the Q and I^2 statistics.
- For exposure categories with substantial methodological heterogeneity or sparse data, findings were synthesized narratively rather than pooled.

12. Subgroup and sensitivity analyses

Planned analyses included:

- By study design (case–control vs. cohort).
- By exposure category (ETS, cooking oil fumes, and solid fuels/biomass).
- Sensitivity analyses were planned and performed to evaluate the stability of pooled estimates, including sequential exclusion of individual studies (leave-one-out approach) and additional excluding studies with lower precision or higher risk of bias.
